# Supplementary material for: A New Derivative of Retro-2 Displays Antiviral Activity against Respiratory Syncytial Virus
Source: Int J Mol Sci. 2023 Dec 28;25(1):415. doi: 10.3390/ijms25010415 (PMC10778932; doi:10.3390/ijms25010415)
Supplement: Supplementary file 1 [file ijms-25-00415-s001.zip › ijms-2708916-supplementary.pdf]

Supplemental Materials

A New Derivative of Retro-2 Displays Antiviral Activity against Respiratory Syncytial Virus

Adrien Le Rouzic [1,2], Jenna Fix [1], Robin Vinck [2,3], Sandrine Kappler-Gratias [4], Romain Volmer [5], Franck Gallardo [4], Jean-François Eléouët [1], Mathilde Keck [2], Jean-Christophe Cintrat [3], Julien Barbier [2], Daniel Gillet [2,*] and Marie Galloux [1,*]

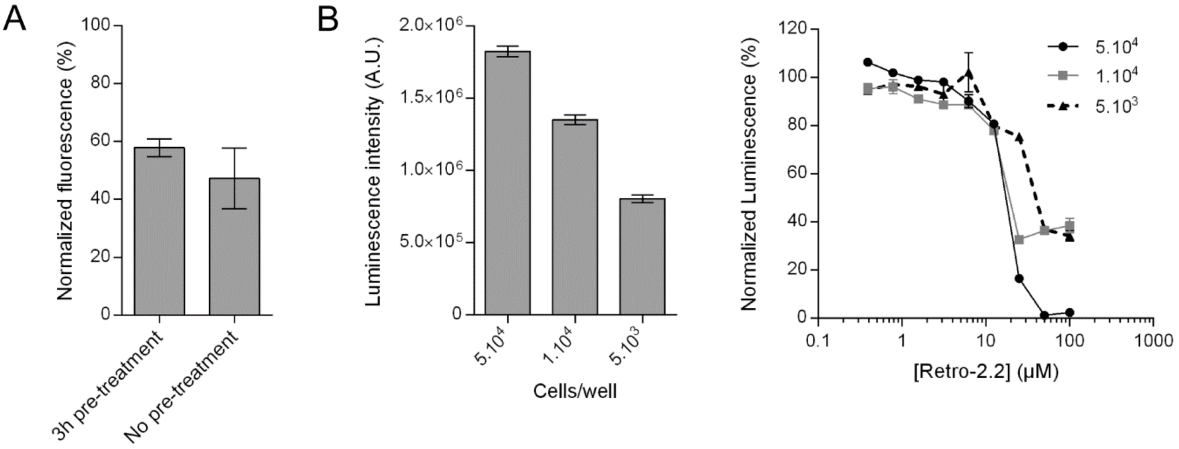

Figure S1. Antiviral activity against hRSV and toxicity of Retro-2.2 on HEp-2 cells. (A) Cells were treated or not for 3 h in the presence of Retro-2.2 at 2.5 μM, before infection with mCherry-rRSV at MOI 0.2. The medium was then changed to incubate cells in the presence of Retro-2.2 at 2.5 μM for 48 h. The viral replication was quantified by measurement of the mCherry fluorescence. Data are means SEM from two independent experiments made in triplicates. **(B)** HEp-2 cells plated at 5.10^4, 1.10^4, or 5.10^3 cells per well of 96-well plates were incubated for 48 h in the presence of serial dilutions of Retro-2.2. Cell viability of non-treated cells (left panel) or cells treated with Retro-2.2 (right panel) was quantified by luminescence, using the CellTiter-Glo luminescent cell viability kit (Promega). Data of treated cells were normalized by the signals of the corresponding control cells. Error bars are standard deviations from duplicates. Data are representative of two experiments.

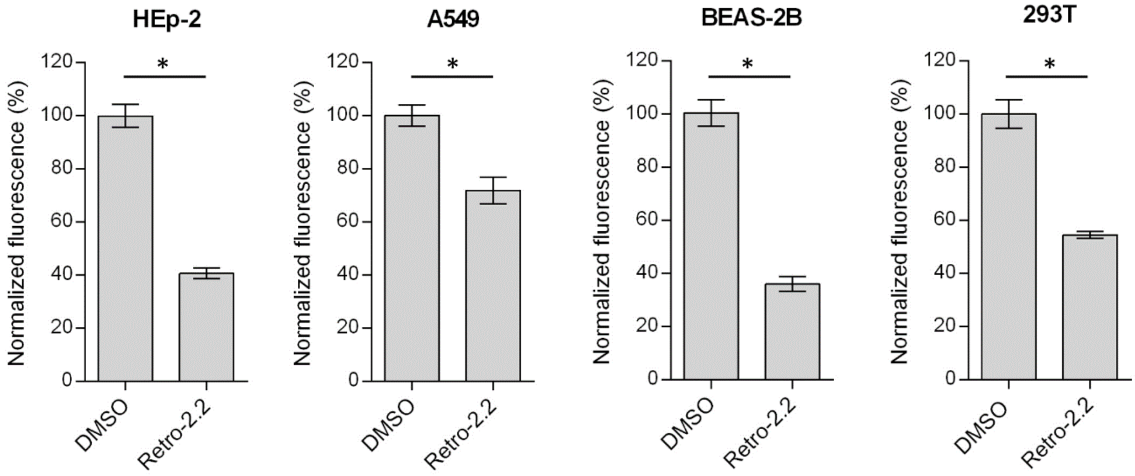

Figure S2. Antiviral activity of Retro-2.2 against hRSV infection on different cell lines. HEp-2, A549, BEAS-2B, and 293T cells were infected for 2 h with mCherry-rRSV at MOI 0.2. The medium was then changed to incubate cells in the absence (DMSO) or the presence of Retro-2.2 at 3 μM for 48 h. The viral replication was quantified by measurement of the mCherry fluorescence. Data are means SEM from two independent experiments made in duplicates. *, $P < 0.05$.
